# Genomic and clinical predictors of lacosamide response in refractory epilepsies

**DOI:** 10.1002/epi4.12360

**Published:** 2019-09-25

**Authors:** Sinéad B. Heavin, Mark McCormack, Stefan Wolking, Lisa Slattery, Nicole Walley, Andreja Avbersek, Jan Novy, Saurabh R. Sinha, Rod Radtke, Colin Doherty, Pauls Auce, John Craig, Michael R. Johnson, Bobby P. C. Koeleman, Roland Krause, Wolfram S. Kunz, Anthony G. Marson, Terence J. O'Brien, Josemir W. Sander, Graeme J. Sills, Hreinn Stefansson, Pasquale Striano, Federico Zara, EPIGEN Consortium, EpiPGX Consortium, Chantal Depondt, Sanjay Sisodiya, David Goldstein, Holger Lerche, Gianpiero L. Cavalleri, Norman Delanty

**Affiliations:** ^1^ School of Pharmacy and Biomolecular Sciences Royal College of Surgeons Dublin Ireland; ^2^ Luxembourg Centre for Systems Biomedicine University of Luxembourg Esch‐sur‐Alzette Luxembourg; ^3^ Department of Neurology and Epileptology Hertie Institute for Clinical Brain Research University of Tübingen Tübingen Germany; ^4^ Centre for Human Genome Variation Duke University Durham NC USA; ^5^ Department of Clinical and Experimental Epilepsy UCL Queen Square Institute of Neurology London UK; ^6^ Chalfont Centre for Epilepsy Buckinghamshire UK; ^7^ School of Medicine Trinity College Dublin Dublin Ireland; ^8^ Department of Neurology St James's Hospital Dublin Ireland; ^9^ Department of Molecular and Clinical Pharmacology Institute of Translational Medicine University of Liverpool Liverpool UK; ^10^ Department of Neurosciences Belfast Health and Social Care Trust Belfast UK; ^11^ Division of Brain Sciences Imperial College Faculty of Medicine London UK; ^12^ Center of Molecular Medicine University Medical Center Utrecht Utrecht The Netherlands; ^13^ Institute of Experimental Epileptology and Cognition Research and Department of Epileptology University of Bonn Bonn Germany; ^14^ The Departments of Neuroscience and Neurology The Alfred Hospital Monash University Victoria Australia; ^15^ Stichting Epilepsie Instellingen Nederland (SEIN) Heemstede The Netherlands; ^16^ deCODE Genetics/Amgen Inc Reykjavik Iceland; ^17^ Pediatric Neurology and Muscular Diseases Unit DINOGMI‐Department of Neurosciences, Rehabilitation, Ophthalmology, Genetics, Maternal and Child Health Institute "G. Gaslini" University of Genova Genova Italy; ^18^ Laboratory of Neurogenetics and Neuroscience Institute G. Gaslini Genova Italy; ^19^ Department of Neurology Hôpital Erasme Université Libre de Bruxelles Brussels Belgium; ^20^ Institute for Genomic Medicine Columbia University New York NY USA; ^21^ The FutureNeuro SFI Research Centre Royal College of Surgeons in Ireland Dublin Ireland; ^22^ Division of Neurology Beaumont Hospital Dublin Ireland

**Keywords:** GWAS, lacosamide, pharmacogenomics, pharmacoresistance, refractory

## Abstract

**Objective:**

Clinical and genetic predictors of response to antiepileptic drugs (AEDs) are largely unknown. We examined predictors of lacosamide response in a real‐world clinical setting.

**Methods:**

We tested the association of clinical predictors with treatment response using regression modeling in a cohort of people with refractory epilepsy. Genetic assessment for lacosamide response was conducted via genome‐wide association studies and exome studies, comprising 281 candidate genes.

**Results:**

Most patients (479/483) were treated with LCM in addition to other AEDs. Our results corroborate previous findings that patients with refractory genetic generalized epilepsy (GGE) may respond to treatment with LCM. No clear clinical predictors were identified. We then compared 73 lacosamide responders, defined as those experiencing greater than 75% seizure reduction or seizure freedom, to 495 nonresponders (<25% seizure reduction). No variants reached the genome‐wide significance threshold in our case‐control analysis.

**Significance:**

No genetic predictor of lacosamide response was identified. Patients with refractory GGE might benefit from treatment with lacosamide.

**Key Points**

- We assessed the pharmacogenomics of lacosamide response in a large cohort of people with refractory epilepsy
- No genomic factors predicting response to lacosamide were identified
- It is possible that lacosamide could be an option for some patients with refractory GGE.

## 1 INTRODUCTION

About 25 antiepileptic drugs (AEDs) are available for the treatment of patients with epilepsy. Biomarkers to predict treatment success for specific AEDs are, however, missing. Clinicians base their choice of AEDs on factors such as syndrome, age, gender, co‐medications, comorbidities, and potential side effects. Despite the increasing number of AEDs in recent years, up to one third of patients with epilepsy remain refractory to antiepileptic treatment and continue to experience seizures.^1^ AEDs are associated with multiple adverse drug reactions (ADRs), such as cutaneous reactions, psychosis, electrolyte imbalance, and weight gain, which often limit their use in clinical practice. The idea of pharmacogenetic biomarkers to predict pharmacoresponse or adverse drug reactions is attractive. To date, however, reproducible discoveries in epilepsy pharmacogenetics are limited to mild to severe cutaneous ADRs to different sodium channel blockers. Polymorphisms in the HLA genes (HLA‐B15:02, HLA‐A31:01),^2^, ^3^ cytochrome P450 genes (CYP2C9*3),^4^ and complement factors (CFHR4)^5^ have been shown to be associated with these adverse reactions.

Lacosamide (LCM) was first licensed in Europe in 2008 and in the United States of America in 2009 for the treatment of focal‐onset seizures. LCM differs from other AEDs, such as carbamazepine, phenytoin, and lamotrigine, in that it is not a “traditional” sodium channel blocker. LCM enhances the slow inactivation of voltage‐gated sodium channels, resulting in the stabilization of hyperexcitable neuronal membranes and the inhibition of repetitive neuronal firing.^6^ For LCM, there are currently no pharmacogenetic data.

We report the findings of an observational study of LCM response in a large cohort of patients with epilepsy. We sought to identify the clinical predictors of response to LCM and to examine genetic variation in the predictions.

## 2 MATERIALS AND METHODS

### 2.1 Recruitment and phenotypic information: EPIGEN cohort

Ethical approval was obtained from the ethics committee of each referral center. Informed consent was obtained from all patients or where applicable, from their legal guardians, during routine clinic attendance.

An initial cohort of 483 patients was used to investigate the clinical predictors of LCM response. All patients were undergoing pharmacological treatment for refractory epilepsy. This was defined as having ongoing seizures despite treatment (current or prior) with two or more appropriate antiepileptic drugs at adequate doses.^7^ Participants were recruited from four tertiary epilepsy referral centers: Beaumont Hospital and St. James’ Hospital, Dublin, Ireland (Royal College of Surgeons in Ireland); the National Hospital for Neurology & Neurosurgery (from the Queen Square and Chalfont sites), London, UK (UCL); Erasmus Hospital, Brussels, Belgium (Université Libre de Bruxelles); and Duke University Medical Centre, North Carolina, USA (Duke University). Patients were excluded if (a) they had a history of chronic alcohol or drug abuse within the previous three years and (b) they were suffering from any other clinically significant disease (eg, cancer, heart failure, and progressive neurological disorder).

This was an observational retrospective study, and patients were seen at varying intervals according to clinical need at individual study sites. It thus was a "real‐life" clinical study where patients were managed according to detailed epileptology analysis at tertiary referral epilepsy centers, and patients were followed on the basis of routine clinical practice and need. Factors, such as determination of epilepsy syndrome classification, seizure frequency, and titration of drug, were determined by the treating epileptologist at each study site.

Phenotypic information including gender, maintenance dose of LCM, syndromic diagnosis, and presence or absence of any reported ADR was recorded. Syndrome diagnosis, based on the 2017 International League Against Epilepsy (ILAE) classification,^8^ was recorded into four separate categories: (a) focal epilepsy, (b) developmental and epileptic encephalopathies (DEE), (c) genetic generalized epilepsy (GGE), and (d) unclassifiable epilepsy.

Response to LCM was based on seizure frequency during treatment compared to baseline frequency. Baseline frequency was calculated as the average number of seizures per month in the three months prior to commencement of LCM. Patients were monitored for response to LCM over an 18‐month period. Seizure frequency was calculated as the average number of seizures per month during LCM treatment. All seizure types were taken into consideration when calculating the seizure frequency. Response to LCM was calculated by comparing the seizure frequency during treatment to the baseline frequency.

Lacosamide response was divided into one of four categories: no response, seizure worsening, greater than 75% reduction in seizure frequency, and seizure freedom. We designed these categories to capture the major profiles of response typically seen in the clinic setting.

1. “Seizure freedom” was assigned to subjects who experienced no seizures for a minimum of 12 months while taking LCM.
2. Subjects who experienced a reduction in seizure frequency of greater than 75% while on LCM treatment were assigned to the “greater than 75% reduction in seizure frequency” category.
3. “No response” was assigned to those for whom the treating clinician felt there was no or little change in seizure frequency while on LCM compared to before treatment, that is, less than 25% seizure reduction.
4. “Seizures worsening” was assigned to those who experienced an increase in seizure frequency during treatment with LCM, that is, greater than 50% increase in seizure frequency.

### 2.2 Recruitment and phenotypic information: EpiPGX cohort

For the subsequent genetic analysis, 183 subjects from the EpiPGX study were included into the cohort (Table 3). EpiPGX is a European‐wide epilepsy research partnership under the European Commission Seventh Framework Protocol (FP7). Patients were recruited from five tertiary epilepsy referral centers: Institute "G. Gaslini", University of Genova, Genoa, Italy; The Walton Centre NHS Foundation Trust, Liverpool, UK; Epilepsy Unit, West Glasgow ACH‐Yorkhill, UK; the University Hospital Bonn, Bonn, Germany; and the University Hospital Tübingen, Tübingen, Germany.

These additional 183 study subjects come from independent nonoverlapping recruitment sites that comprise the EpiPGX study. These were included as an independent cohort to increase the study numbers and thus increase the power to detect potential genomic differences between the extremes of lacosamide responders (greater than 75% seizure reduction) on the one hand and nonresponders (less than 25% reduction) on the other.

### 2.3 Testing for genetic predictors of response

Genotype data were available on 570 patients. Samples were genotyped either at Duke University Centre for Human Genome Variation (NC, USA) on the Illumina Human610 beadchip or at DeCODE genetics (Reykjavik, Iceland) on the Illumina HumanOmniExpress beadchip platform. Imputation to the phase 1v3 (March 2012) 1000 Genomes reference panel was performed as follows: genotype data were filtered to remove SNPs with low call rate (>5% missingness), Hardy‐Weinberg equilibrium (*P* < 10^−6^), and <1% minor allele frequency (MAF). Duplicates were removed while gender mismatches were checked and errors resolved. Using this subset of markers, heterozygosity and identity by state (IBS) were calculated in order to remove all samples with outlying heterozygosity values (>5 standard deviations from the median of the whole sample) and one half of all sample pairs with >0.9 IBS. Sample ethnicity was assessed through STRUCTURE,^9^ and only samples matching European ancestry were included (CEU > 90%, as determined from HapMap populations). Genotype data were aligned to positive strand and separated by chromosome prior to phasing with SHAPEIT.^10^ Phased chromosomes were imputed to the 1000 Genomes phase 1v3 reference panel with IMPUTE2.^11^ Imputed variants were filtered to only those with high call rate (>0.9), high confidence score (info > 0.95), and common MAF (>2%). Genotype data were cleaned using standard quality control metrics with PLINK,^12^ GCTA,^13^ and GTOOL.

We also investigated the contribution of rarer genetic variation from whole exome sequencing data within the same case‐control response groups above. A total of 110 samples were sequenced at Duke University Centre for Human Genome Variation (NC, USA) using the Roche Nimblgen SeqCap EZ Exome target enrichment platform. A further 66 samples were sequenced at DeCODE genetics (Reykjavik, Iceland) using the Illumina Nextera target enrichment platform. Individual FASTQ files were aligned to human genome reference b37 with Burrows‐Wheeler Aligner. Resultant BAM files were then processed through the GATK Best Practices pipeline to remove duplicate reads, align indels, and recalibrate base quality scores to generate individual genomeVCF files. The cohort of genomeVCFs was then joint‐genotyped into a single multisample VCF file. Variant annotation was performed with ANNOVAR. Only sites with minimum 10× coverage across all subjects were considered for analysis.

### 2.4 Statistical methods

Multiple logistic regression of clinical covariates was used to test for gender and ILAE diagnosis as predictors of treatment outcome. A logistic regression model was used to test dose as a predictor of treatment outcome and to test gender as a predictor of reported ADR. Statistical analysis was performed with the use of Stata^®^ (version IC 13) software package.

We defined cases as any subject with a broadly positive response (greater than 75% seizure reduction or seizure freedom), while controls were defined as those with no response or seizures worsening.

We performed a primary case‐control association test in PLINK using a logistic regression model, with syndromic diagnosis and five principal components as covariates, pooling samples from EPIGEN and EpiPGX. We subsequently performed the following secondary subanalyses: seizure freedom vs no response, greater than 75% reduction of seizure frequency vs no response, and seizures worsening vs no response. Drug dosage data were missing in >10% of samples and were therefore not included as a covariate. A minimum minor allele frequency threshold of 2% was applied to genotype data. We estimated that the study had 80% power to detect a genetic predictor of relative risk (approximated to odds ratio) ≥ 6 with an allele frequency ≥ 2% and an alpha level of 5 × 10^−8^. The study power improves with increasing minor allele frequency (Figure S2).

In our exome analysis, we performed the same set of primary and secondary case‐control analyses as described above. We included any functional variant (missense, nonsense, frameshift, and splice site variants) with a maximum population‐wide minor allele frequency of 2% in gnomAD. When analyzing, we collapsed qualifying variants per gene and performed a SKAT‐O test with syndromic diagnosis, sequencing site, and five principal components as covariates in the model. We considered a total of 281 genes composed of 267 genes involved in absorption, distribution, metabolism and excretion (ADME) and 14 sodium channel genes (the full list of genes can be found in Table S1). We excluded genes with less than two polymorphic variants in our cohort. We applied a Bonferroni corrected significance threshold of *P* = 5.9 × 10^‐05^ to account for the 281 genes assessed in the three logistic regression models listed above.

## 3 RESULTS

### 3.1 Clinical predictors

A total of 483 patients (276 females) were included in the study from four centers as part of the EPIGEN consortium. The majority of them (n = 479) were treated with LCM in addition to other AEDs.

The majority of patients had focal epilepsy (91%), while 4% had genetic generalized epilepsy, 3% had developmental and epileptic encephalopathies, and 2% had an unclassifiable epilepsy diagnosis (Table 1).

**Table 1:** Breakdown of syndromic epilepsy diagnosis and response categories for each of the four tertiary referral centers

	Dublin	London	Brussels	North Carolina	Combined cohort
Epilepsy diagnosis					
Focal epilepsy of known etiology	51 (57%)	177 (72%)	31 (61%)	47 (49%)	306 (64%)
Focal epilepsy of unknown etiology	29 (33%)	52 (21%)	18 (35%)	33 (35%)	132 (27%)
Developmental and/or epileptic encephalopathies	5 (6%)	8 (3%)	0	3 (3%)	16 (3%)
Genetic generalized epilepsy	3 (3%)	6 (2%)	0	9 (9%)	18 (4%)
Unclassifiable epilepsy	1 (1%)	4 (2%)	2 (4%)	4 (4%)	11 (2%)
Total	89	247	51	96	483 (100%)
Response categories					
Seizure freedom	3 (3%)	3 (1%)	3 (6%)	9 (9%)	18 (4%)
≥75% reduction in seizure frequency	10 (11%)	8 (3%)	5 (10%)	22 (23%)	45 (9%)
No response	65 (74%)	219 (89%)	35 (69%)	63 (66%)	382 (79%)
Seizures worsening	11 (12%)	17 (7%)	8 (15%)	2 (2%)	38 (8%)
Total	89	247	51	96	483

Note

Percentage of each syndrome/ response category for each site and the combined cohort are shown in parenthesis.

The response categories for each tertiary referral center are shown in Table 1. Seizure freedom rate ranged from 1% to 9% across the four referral centers, with an average of 4% for the combined cohort. Overall, 13% of patients had a positive response to LCM treatment (seizure freedom or greater than 75% reduction in seizure frequency). Eight percent had an increase of seizures during LCM treatment (range 2%‐15%), while 79% of patients showed no response to LCM treatment (range 66%‐89%).

**Figure 1:**
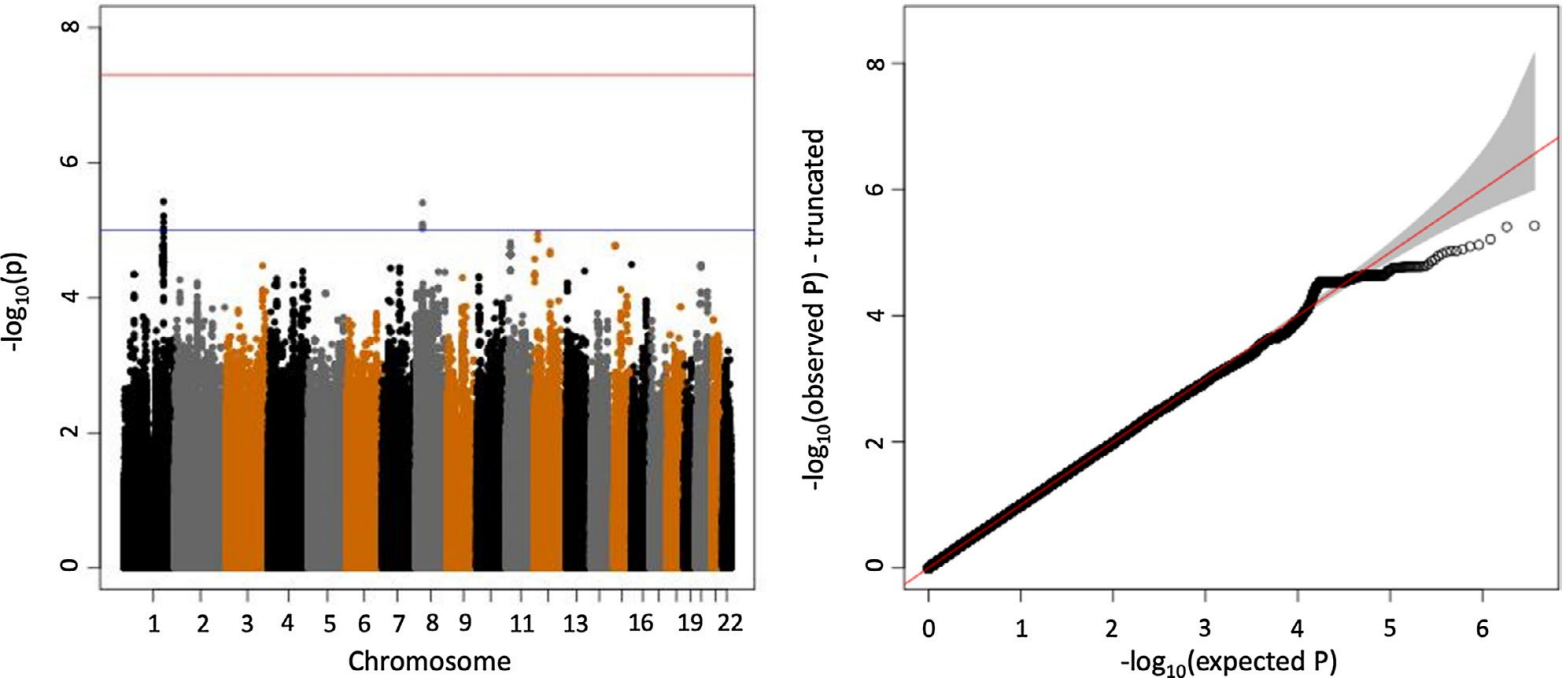
Manhattan plot and quantile‐quantile plot for genome‐wide association analysis of broad response vs no response or seizures worsening [Genomic Inflation Factor = 1.01]

Maintenance dose ranged from 25 to 800 mg/d, with the majority (n = 139) maintained on 400 mg/d of LCM (Figure 1A). The average maintenance dose was compared across the four response categories (Figure 1B). Patients who had seizure aggravation while on LCM had the lowest average maintenance dose compared to the other response groups. Patients who had a greater than 75% reduction in seizure frequency had the highest average maintenance dose.

#### 3.1.1 Gender as a predictor of response

To test whether gender was a significant predictor of response to LCM treatment, we developed a logistic regression model with response as the dependent variable, and gender and ILAE syndromic diagnosis as the predictor variables. For each test, the specific response category of LCM treatment (seizures worsening/greater than 75% reduction in seizure frequency/seizure freedom) was compared to the category of “no response to LCM treatment.” Gender did not emerge as a significant predictor for any of the response categories (seizures worsening, *P* = .225; greater than 75% reduction in seizure frequency, *P* = .994; seizure freedom, *P* = .073).

#### 3.1.2 Syndromic diagnosis as a predictor of response

As LCM is licensed for the treatment of focal‐onset seizures, we wished to test whether other syndromic categories (ie, GGE, DEE, and unclassifiable) responded in a different way compared to the “focal” category. We therefore compared response profiles of each of the generalized and unclassified groups, to focal (known and unknown etiology) epilepsy. To test diagnosis as a predictor of LCM response, each of the response categories (seizures worsening/greater than 75% reduction in seizure frequency/seizure freedom) was compared to the “no response” category. Syndromic diagnosis was taken into consideration for each of these response comparisons (Table 2). The results suggest that patients with GGE were five times more likely to respond to treatment with LCM, when compared to patients with a focal diagnosis (*P* = .049). Syndromic diagnosis did not emerge as a significant clinical predictor for patients who experienced a worsening seizure activity following treatment with LCM.

**Table 2:** Diagnosis as a predictor of LCM response

Response	Diagnosis	n	RRR (95% CI)	*P*
Seizure freedom	DEE	0	na	na
	GGE	2	**5.06 (1.01‐25.22)**	.049*
	Unclassifiable epilepsy	2	**8.42 (1.55‐45.67)**	.014*
Greater than 75% reduction in seizure frequency	DEE	2	1.60 (0.34‐7.44)	.548
	GGE	4	**4.24 (1.24‐14.42)**	.021*
	Unclassifiable epilepsy	1	1.73 (0.20‐14.91)	.618
Seizures worsening	DEE	1	0.79 (0.98‐6.44)	.830
	GGE	2	1.73 (0.35‐8.60)	.500
	Unclassifiable epilepsy	1	3.45 (0.64‐18.52)	.149

Note

Focal epilepsy was used as the base variable for diagnosis. Response to LCM treatment (seizures worsening/≥75% reduction in seizure frequency/seizure freedom) was compared to the “no response” category. No patient with DEE achieved seizure freedom (na = not applicable). Values that reached significance (*P < .05) are shown in bold.

Abbreviations: CI, confidence interval; RRR, relative risk ratio for variables associated with LCM response.

### 3.2 Genomic predictors

Genotype data were available on 570 patients for the GWAS analysis; 387 patients from the EPIGEN cohort; and a further 183 patients from the EpiPGX cohort. Following genotype imputation and quality control, there were genotype dosage data for 5,205,884 SNP markers. Table 3 shows the breakdown of response categories for both cohorts.

**Table 3:** Sample count for lacosamide response categories in EPIGEN and EpiPGX

Response	EPIGEN	EpiPGX	Total GWAS	Total WES
Seizure freedom	15	14	29	25
Greater than 75% reduction in seizure frequency	31	15	46	14
No response	305	143	448	90
Seizures worsening	36	11	47	31
Subtotal	387	183	570	160

Abbreviation: WES, whole exome sequencing.

To test for association with broad response to LCM, we carried out a genome‐wide analysis of the broad response group (n = 75) compared to patients with no response or seizures worsening (n = 495). We did not detect any variants satisfying our threshold for genome‐wide significance (Figure 1). Further comparisons between smaller groups also did not yield significant results (see Figure S3).

**Figure 2:**
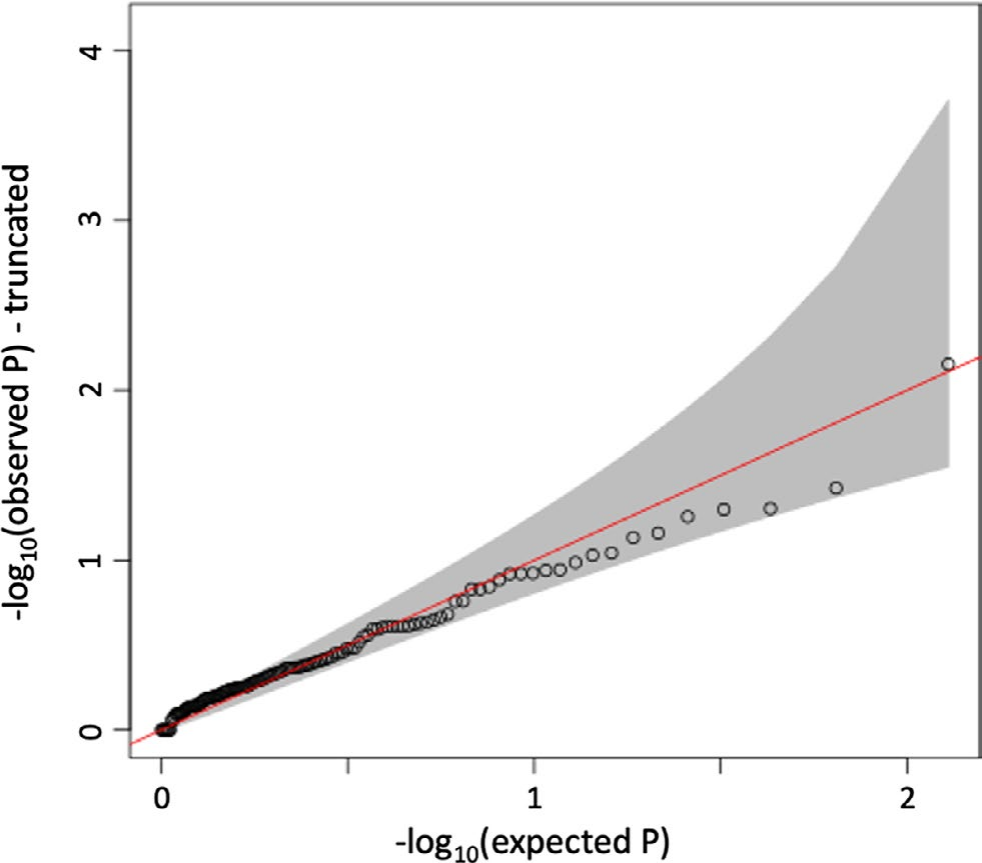
Q‐Q plot of SKAT‐O test of candidate gene‐set association with LCM response

Following quality control of exome sequences, there were 160 subjects available for analysis. The results from our SKAT‐O tests did not reveal a significant contribution of rare variants to LCM response after correction for multiple testing (Figure 2 and Table 4). The full set of test results can be found in the supplements.

**Table 4:** Top SKAT‐O results for gene‐set analyses of each response group

Analysis	Gene	No. SNV	Q	Rho	*P*
Broad response vs no response	*CYP24A1*	2	426.474	1	.007
	*SLC22A16*	2	597.149	0	.037
	*CYP3A7*	2	247.833	0	.049
	*ALDH9A1*	4	606.731	0	.050
	*FMO3*	2	433.141	1	.055
	*GSTM5*	2	565.228	1	.069

Abbreviations: Q, SKAT test statistic; *P*, uncorrected significance value; Rho, 0 or 1 for SKAT or burden test, respectively; SNV, number of polymorphic markers in gene.

## 4 DISCUSSION

In our large cohort, 4% became seizure‐free following treatment with LCM, similar to that seen previously, where 3.4% of subjects had achieved seizure freedom at three months of evaluation.^14^ Most studies, including the three regulatory randomized controlled trials, evaluated the number and proportion of subjects who had a 50% reduction in seizure frequency, known as 50% responders 15, 16, 17, 18, 19, 20 (reviewed in Ref.^21^), and reported between 18% and 69% of subjects meeting this criterion.^21^ We evaluated response as greater than 75% reduction in seizure frequency (to enrich the partial response group for genetic analysis), and thus, comparison with other studies is not possible for this response group. Nonresponders accounted for up to a third of subjects in previous studies.^21^ Our subcohorts of nonresponders (no response and seizure worsening) accounted for 87% of our cases. This difference could be explained by the fact that our cohorts were derived from tertiary referral centers which handle many patients with highly refractory epilepsies. As in this study, previous studies reported few subjects that had seizure aggravation.^14^ In our cohort, the worsening cases had the lowest average LCM dose, some cases even below 100 mg. It is probable that seizure aggravation was due to other unknown factors or was just observed by chance as result of natural fluctuation of seizure frequency and was not an immediate consequence of LCM treatment.

This study looked at gender and syndrome diagnosis as clinical predictors of response. Gender was not predictive of LCM response in our data. This finding, together with the evidence that gender does not affect the pharmacokinetic profile of LCM,^22^ suggests gender is not playing a role in the varying response of patients to LCM. Many studies have evaluated the efficacy and safety of LCM in adults with refractory epilepsy and focal seizures only (reviewed in Paquette *et al* 15). In a separate study evaluating the safety and efficacy of LCM in both pediatric and adult populations, patients with focal and generalized epilepsy were included.^14^ The clinical outcome results in our patients with refractory focal epilepsy contrast with another real‐world multisite observational study from Germany of the use of lacosamide added to one prior antiepileptic drug in 520 patients, in which 63.8% of patients had a greater than 75% seizure reduction, reflecting a presumably much less refractory population.^23^ While LCM has been shown to be effective in focal epilepsy and status epilepticus, the use of LCM in the treatment of refractory GGE is limited to smaller studies and case series but showed rather positive results.^19^, ^24^, ^25^, ^26^ Patients with GGE responded well to LCM treatment compared to those with focal epilepsy (Table 3). Our cohort of patients with GGE is small, and our study was observational, unblended, and not designed to test efficacy by syndrome. Therefore, these results have to be considered with caution. Nonetheless, they are suggestive that patients with GGE may benefit from treatment with LCM and are in line with previous studies.

Genome‐wide meta‐analysis did not identify a significant contribution of common or rare genetic variants to lacosamide response satisfying our threshold for significance, for any of the response groups (Figures 1, 2) despite 80% power to detect a genetic predictor of relative risk (approximated to odds ratio) ≥ 6 with an allele frequency ≥ 2%. These findings underline that pharmacoresistance constitutes a complex trait that is not solely driven by one or a few genetic factors. Larger cohorts of patients could help to further elucidate this question. More novel analysis techniques such as the polygenic risk score^27^ or the polygenic transmission disequilibrium test^28^ could also help to elucidate the role of common variants in future studies.

A limitation of our study is that it is a biased observational study in patients with refractory disease attending tertiary referral centers. However, refractory patients who may respond to a newly introduced AED such as lacosamide might have novel pharmacogenomic factors that may be identifiable even in a relatively small cohort of patients. In this type of study in a refractory cohort, a small group of patients with previously drug‐resistant epilepsy who become seizure‐free or whose seizure control markedly improves when another new drug is added to their treatment regimen (a group that might be termed “unexpected responders”) might be a cohort in which we might gain important pharmacogenomic response insights. We believe that our study is an important proof‐of‐principle pharmacogenomic approach that could be adopted by the epilepsy clinical trial community and industry partners to incorporate into future randomized controlled trials of new AEDs. Therefore, further such pharmacogenomic response studies would also ideally be performed in either new‐onset patients or patients earlier in the course of their epilepsy.

## CONFLICT OF INTEREST

This work was funded by an Investigator‐Initiated Study award from UCB‐Pharma. We confirm that we have read the Journal's position on issues involved in ethical publication and affirm that this report is consistent with those guidelines.

## AUTHOR CONTRIBUTIONS

Please list the contributions of each author to the piece; please refer to the Author Disclosure Form for our authorship criteria.

## INFORMATION PERTAINING TO WRITING ASSISTANCE

N/A

## APPENDIX 1

**EPIGEN Centres**: Beaumont Hospital and St. James’ Hospital, Dublin, Ireland (Royal College of Surgeons in Ireland); the National Hospital for Neurology and Neurosurgery, London, UK (University College London); Erasmus Hospital, Brussels, Belgium (Université Libre de Bruxelles); and Duke University Medical Centre, North Carolina, USA (Duke University).

**EpiPGX Banner**: Ingason A, Langley SR, Willis J, Leu C, Heggeli K, Weckhuysen S, Brodie MJ, Stefánsson K, Jorgensen A, Francis B, Srivastava P, Weber Y, Rau S, Becker F, Sonsma A, Krenn M, Zimprich F.
