# Supplementary material for: Genomic and clinical predictors of lacosamide response in refractory epilepsies
Source: Epilepsia Open. 2019 Sep 25;4(4):563–71. doi: 10.1002/epi4.12360 (PMC6885661; doi:10.1002/epi4.12360)
Supplement: Supplementary file 5 [file EPI4-4-0-s005.docx]

**Supplements**

(a)


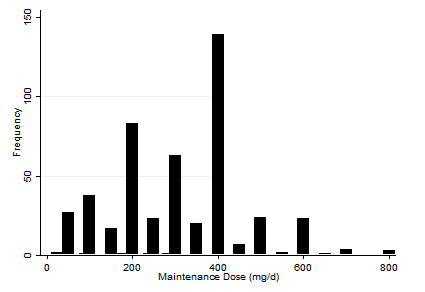


(b)

**Supplement Figure 1** **(a)** Frequency of LCM maintenance dose. **(b)** Average maintenance dose for each of the response groups to LCM treatment.

**
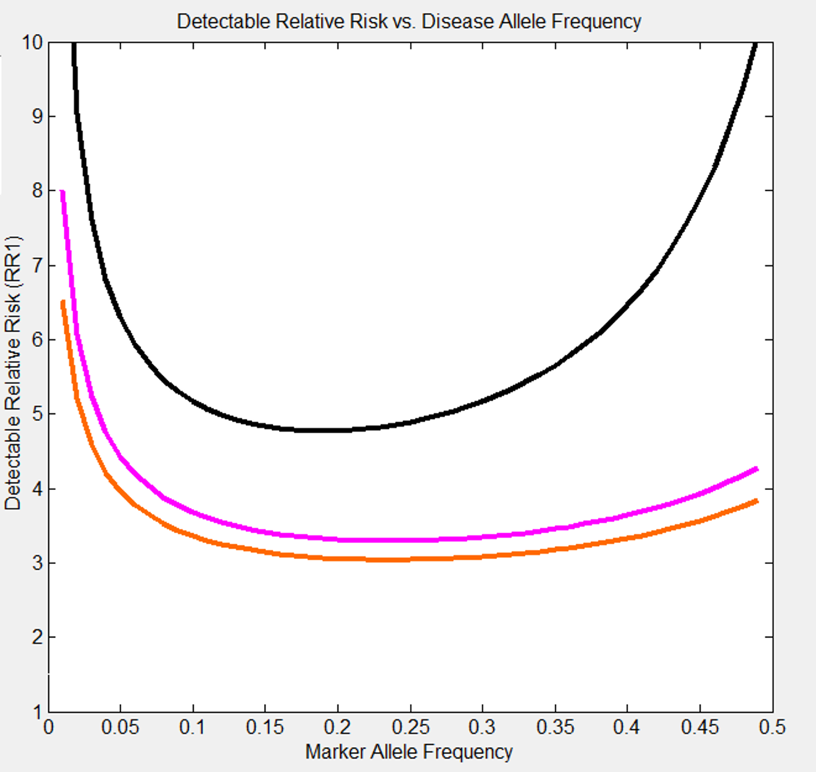
**

**Supplementary Figure 2:** Power curves for GWAS. We estimated to have 80% power to detect a genetic association for seizure freedom (black), >75% seizure reduction (orange) and seizures worsening (pink).

**
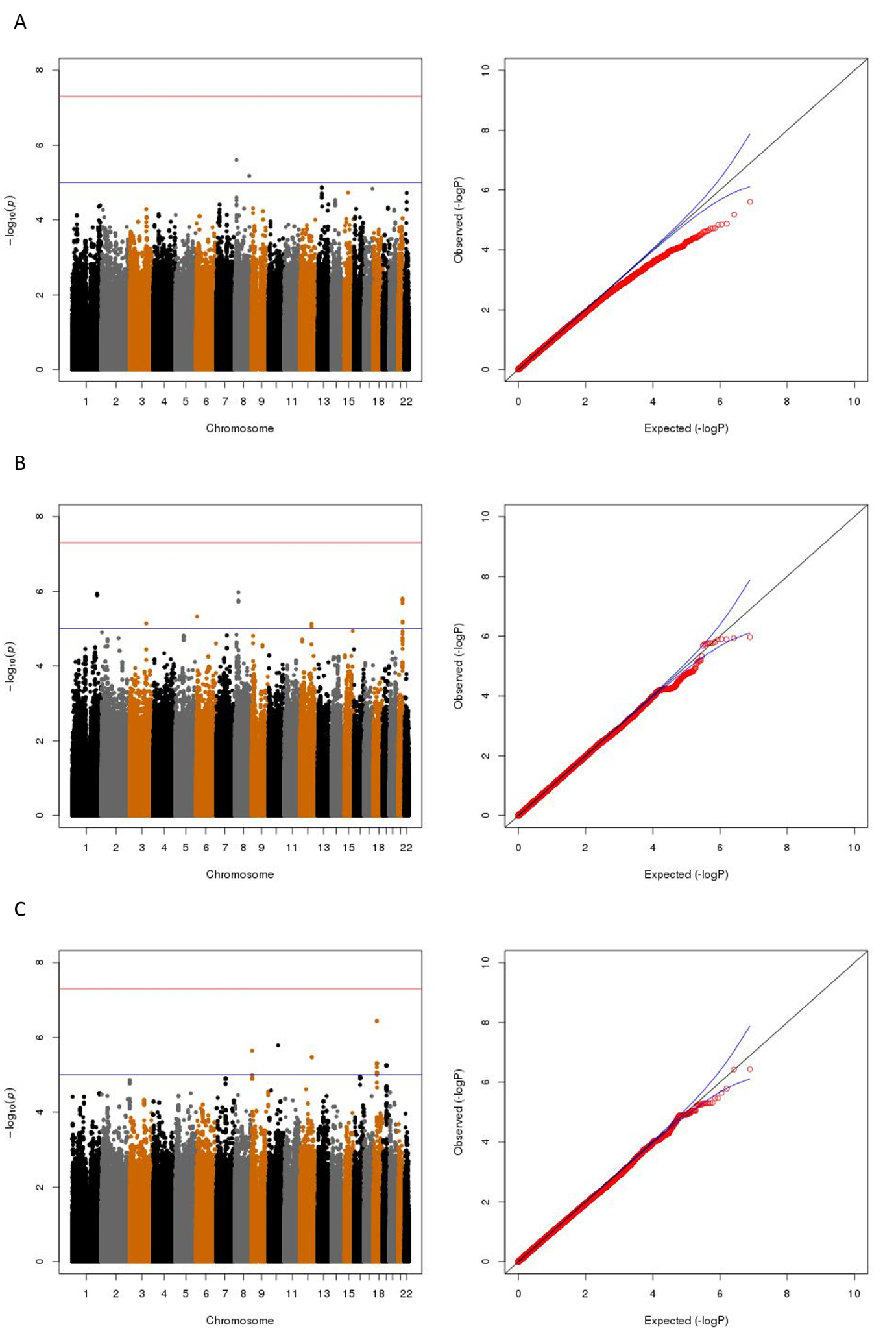
**

**Supplementary Figure 3.** Manhattan plot and quantile-quantile plots for genome-wide association analyses of subgroups; (A) seizure freedom vs no response [Genomic Inflation Factor = 0.995]; (B) ≥75% reduction in seizure frequency vs no response [Genomic Inflation Factor = 0.987]; (C) seizures worsening vs no response; [Genomic Inflation Factor = 0.991].


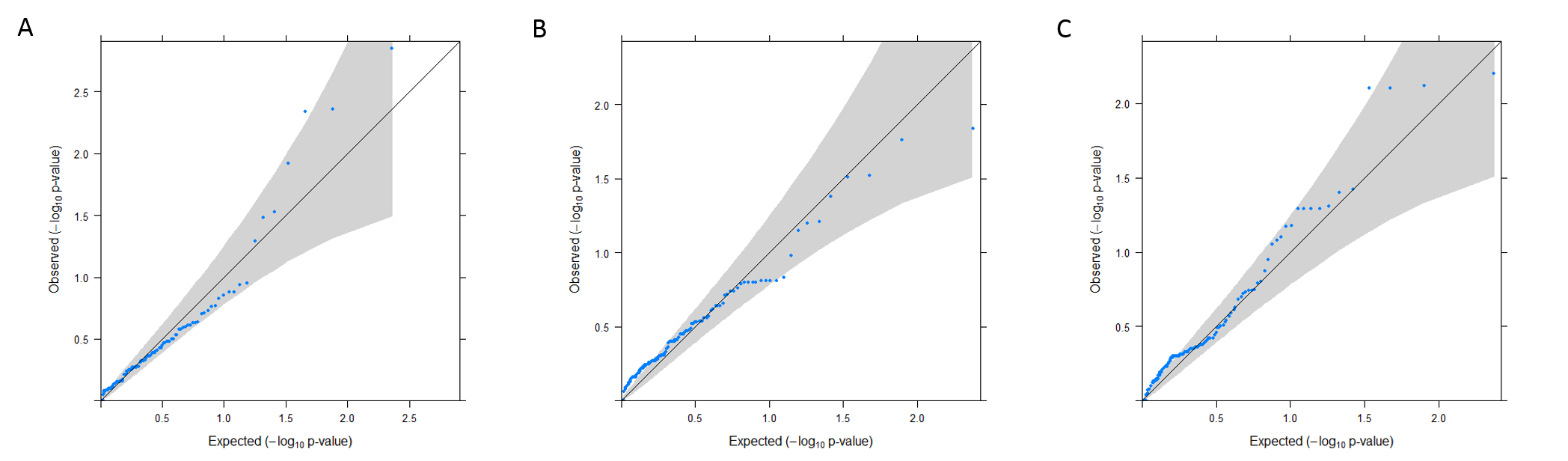


**Supplementary Figure 4:** Q-Q plots of SKAT-O results for gene-set analyses of each response subgroup. (A) seizure freedom vs no response; (B) ≥75% reduction in seizure frequency vs no response; (C) seizures worsening vs no response.

| Analysis | Gene | No. SNV | Q | Rho | P |
| --- | --- | --- | --- | --- | --- |
| Seizure freedom vs no response | *CYP24A1* | 2 | 447.143 | 1 | 0.001 |
|  | *POR* | 5 | 1414.62 | 1 | 0.004 |
|  | *ARNT* | 2 | 380.775 | 0 | 0.004 |
|  | *ADH1B* | 3 | 345.395 | 0.9 | 0.012 |
|  | *SLC22A16* | 2 | 448.312 | 0 | 0.029 |
|  | *CYP3A7* | 2 | 232.564 | 0.6 | 0.032 |
| >75% reduction vs no response | *CYP24A1* | 2 | 367.083 | 1 | 0.014 |
|  | *FMO3* | 2 | 557.357 | 1 | 0.017 |
|  | *SLC22A16* | 2 | 554.989 | 0 | 0.030 |
|  | *POR* | 5 | 1146.1 | 1 | 0.030 |
|  | *ARNT* | 2 | 331.455 | 0 | 0.041 |
| Seizures worsening vs no response | *UGT1A8* | 5 | 1361.8 | 1 | 0.006 |
|  | *GSR* | 2 | 399.046 | 1 | 0.008 |
|  | *UGT1A9* | 4 | 692.353 | 0.9 | 0.008 |
|  | *UGT1A10* | 4 | 692.353 | 0.9 | 0.008 |
|  | *ARNT* | 4 | 821.613 | 1 | 0.037 |
|  | *ABCB8* | 4 | 260.467 | 0.5 | 0.040 |
|  | *CYP8B1* | 2 | 412 | 1 | 0.049 |

**Supplementary Table 1**: Top SKAT-O results for candidate gene-set analyses of each response subgroup.
